# Ultrafast Dynamics of Diketopyrrolopyrrole Dimers

**DOI:** 10.1002/jcc.27547

**Published:** 2024-12-14

**Authors:** Ali Al‐Jaaidi, Josene M. Toldo, Mario Barbatti

**Affiliations:** ^1^ Aix Marseille University, CNRS, ICR Marseille France; ^2^ UCBL, ENS de Lyon, CNRS, LCH, UMR 5182 Lyon Cedex 07 France; ^3^ Institut Universitaire de France Paris France

**Keywords:** charge transfer mechanisms, diketopyrrolopyrrole (DPP), excited‐state dynamics, exciton dynamics, hydrogen migration, organic photovoltaics (OPV), photophysics, surface hopping, time‐dependent density functional theory (TDDFT), ultrafast internal conversion

## Abstract

Diketopyrrolopyrroles (DPPs) have attracted attention for their potential applications in organic photovoltaics due to their tunable optical properties and charge‐carrier mobilities. In this study, we investigate the excited‐state dynamics of a DPP dimer using time‐dependent density functional theory (TDDFT) and nonadiabatic molecular dynamics simulations. Our results reveal a near‐barrierless hydrogen migration state intersection that facilitates ultrafast internal conversion with a lifetime of about 400 fs, leading to fluorescence quenching. Electronic density analysis along the relaxation pathway confirms a hydrogen atom transfer mechanism. These findings highlight the critical role of state intersections in the photophysical properties of DPP dimers, providing new insights for the design of functionalized DPP systems aimed at suppressing nonradiative decay for enhanced performance in photovoltaic applications.

## Graphical abstract

Using nonadiabatic dynamics, we uncover an ultrafast hydrogen migration‐driven internal conversion pathway in diketopyrrolopyrrole dimers with a sub‐picosecond excited‐state lifetime. This work highlights the critical impact of state intersections on photophysical performance, offering insights for functionalizing DPP systems to suppress nonradiative decay.

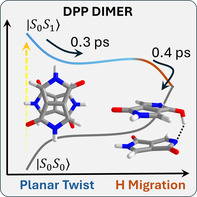

## 1 Introduction

**FIGURE 1:**
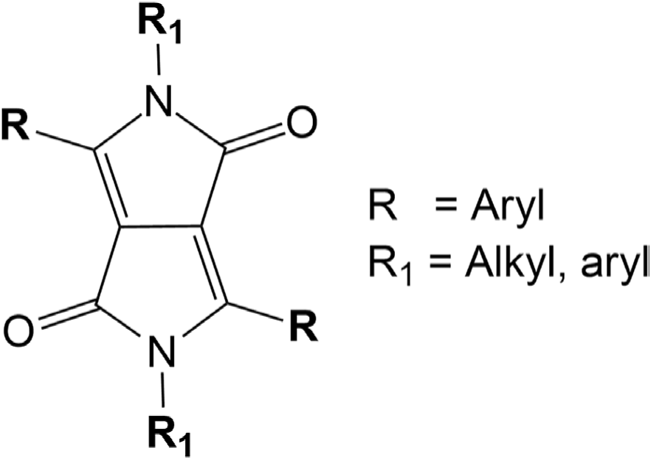
Molecular structure of DPP core and its more common substituents.

As the demand for renewable energy continues to rise, organic photovoltaic (OPV) materials have garnered significant attention due to their tunable properties and cost‐effectiveness [1]. Among these materials, diketopyrrolopyrroles (DPPs; Figure 1) stand out for their versatility, ease of functionalization, and strong absorption characteristics [2]. DPP‐based systems have demonstrated promising power conversion efficiencies (PCEs) in OPVs. Yet their full potential remains limited by certain photophysical behaviors, particularly related to nonradiative decay pathways. Addressing these limitations requires a deeper understanding of the excited‐state dynamics of DPPs at the molecular level.

DPP and its derivatives have been used as dyes due to their strong absorption and facility to increase the conjugation length due to functionalization. They also exhibit interesting intrinsic photophysical properties, such as high fluorescence quantum yields and tunable absorption spectra (up to the 1000 nm region) [3, 4]. Moreover, these cost‐effective pigments show high charge‐carrier mobilities in conjugated systems, excellent crystallinity, and notable thermal and photochemical stability [2].

Since 2008, DPP‐based molecules have been used in OPVs and organic electronics [2, 5, 6]. Their use as building blocks for high‐performance organic semiconductors is promising since the replacement of the functional groups can affordably tune the DPP's absorption spectrum and p‐type, n‐type, or ambipolar character. DPP‐based polymer donors have achieved PCEs of 9.4%, with broad optical absorption and good film‐forming characteristics (good fill factors FF and short‐circuit currents J_SC_) [5]. Currently, the PCE of DPP‐containing small molecule donors reaches around 8% [6]. However, most recently, a significant PCE of 12.0% has been achieved [2]. Besides that, DPP‐based small molecules have been shown to be advantageous over their polymer counterparts due to their easier synthesis and their tendency to self‐assemble, enhancing their charge carrier mobilities. For this reason, understanding exciton transport in DPPs‐aggregates is fundamental to identifying the key parameters correlated with the efficiency of excitation energy transport [7]. Yet, higher open‐circuit voltage *V*_OC_, defined molecular structure, and purification are more manageable with DPP‐containing small molecules. Moreover, DPP derivatives have also been used to stabilize double‐excited states and control internal conversion via manipulation of the side chains [8].

This interest in the multiple DPP functionalities has motivated us to investigate the photophysics of DPP dimers. As we shall discuss, the photophysics of these dimers is particularly complex due to the presence of intersections between the excited and ground states, facilitating ultrafast internal conversion, quenching fluorescence, and reducing exciton and charge transport. Such processes are detrimental to the performance of materials designed for light‐harvesting applications, where long‐lived excited states are crucial. Despite the widespread use of DPP derivatives in OPVs and other organic electronics, the mechanistic details of their excited‐state dynamics—especially in dimeric systems—remain insufficiently explored.

In this study, we investigate the excited‐state dynamics of a DPP‐core stacked dimer using time‐dependent density functional theory (TDDFT) and nonadiabatic molecular dynamics (NAMD) simulations. By examining the pathways leading to internal conversion, we identified critical molecular features that control the photophysical behavior of these systems. Our results reveal a previously unreported near‐barrierless hydrogen‐migration S_1_/S_0_ intersection that drives rapid internal conversion, suggesting a crucial role for molecular functionalization in inhibiting these decay pathways.

## 2 Methods

### 2.1 Electronic Structure

Electronic structure calculations were done for the DPP monomer and DPP dimer. Ground state calculations were done using density functional theory (DFT) with the B3LYP and CAM‐B3LYP functionals [9]. Excited states were computed with the linear‐response TDDFT with the CAM‐B3LYP and its Tamm–Dancoff approximation (TDA) using Turbomole V7.6 [10] and ORCA 5.0.4 software [11]. Calculations were done with the def2‐TZVP and 6‐31G** basis sets. The use of DFT‐based methods was an excellent cost–benefit compromise, as multiple guess structures were tested for DPP dimers in the search for the most stable conformer for the ground state geometry. The resolution of identity (RI) approximation was used as implemented in Turbomole. Long‐range dispersion interactions (van der Waals forces) were accounted for using the DFT‐D4 dispersion correction model [12]. Nudged elastic band (NEB) was used in ORCA 5.0.4 [11, 13] to calculate the minimum energy path between the geometries of interest.

Additional calculations were done using the coupled cluster with approximated second‐order (CC2), algebraic diagrammatic construction to the second‐order (ADC(2)), DFT‐based multireference configuration interactions (DFT/MRCI) [14, 15], and MRCI based on orthogonalization‐ and dispersion‐corrected semiempirical method 3 (ODM3/MRCI) [16]. These benchmark excitation energies are presented in Supporting Information S1.

Electronic density analysis was done using TheoDORE software [17]. Natural transition orbitals (NTOs), charge transfer (CT), and average position of the excitation (POS) descriptors were investigated.

### 2.2 Initial Conditions

**FIGURE 2:**
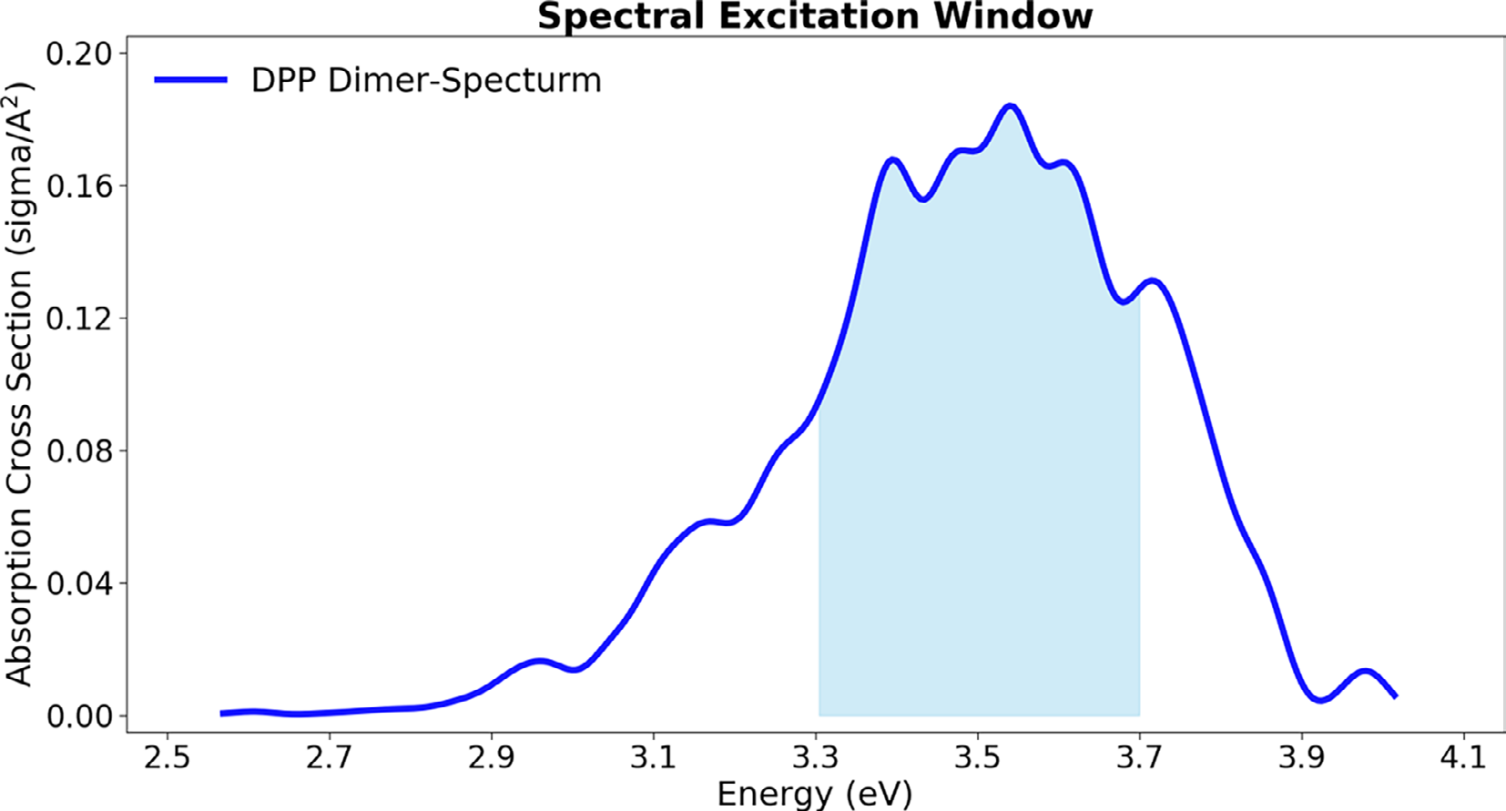
Simulated absorption spectra of DPP dimer calculated using TDA with CAM‐B3LYP/6‐31G**. The shaded area indicates the spectral window from where the initial conditions for the dynamics were selected. With the same computational level, the lowest vertical excitations at the S_0_ minimum occur at 3.53 eV for the degenerated S_1_ and S_2_ states.

The primary purpose of the dynamics was to study the relaxation pathway of the nonfunctionalized DPP dimer after vertical excitation near the excitation band maximum. To do so, 500 initial conditions were generated from a harmonic Wigner distribution around the S_0_‐minimum calculated using B3LYP/6‐31G**. TDA/CAM‐B3LYP/6‐31G** was employed to compute vertical excitation energies and oscillator strengths for the sampled points. The initial conditions and dynamics were generated using the Newton‐X [18] interface to ORCA 5.0.4 [11]. The nuclear ensemble [19] absorption spectrum is shown in Figure 2. The geometries were then filtered by choosing the excitation energy centered at the maximum absorption wavelength (3.5 ± 0.2 eV). This selected window (shaded area in Figure 2) resulted in a ratio of 41:84 accepted geometries for the first and second excited states, respectively. A total of 120 trajectories were generated, obeying the 1:2 proportion between S_1_ and S_2_.

### 2.3 Surface Hopping

The fewest switches surface hopping (FSSH) method was employed, incorporating decoherence effects with the simplified decay of mixing [20] using alpha = 0.1 Hartree. All the dynamics included three states (ground plus two excited states) computed with TDA at CAM‐B3LYP/6‐31G** + D4 level. The TDA approximation was used as it shows a good tradeoff between accuracy and computational cost and may perform better for dynamics than TDDFT [21, 22]. The dynamics were run up to 1 ps, using a classical step size of 0.5 fs; hence, a maximum of 2000 single‐point calculations were performed per trajectory. The time‐dependent Schrödinger equation was integrated with a timestep of 0.025 fs, with electronic properties interpolated between the classical steps.

The reduced kinetic energy protocol [23] (where the kinetic energy is divided by the number of degrees of freedom to evaluate back hoppings) was used. This procedure is discussed in Ref [23] and used to avoid an artificial excess of back hoppings. The momentum remained unchanged in cases of frustrated hoppings. Time‐derivative nonadiabatic couplings were computed with the default options of time‐dependent Baeck–An (TDBA) [24] approximation, which uses the energy gaps between the adiabatic states and their second time‐derivative instead of wavefunctions to estimate the state coupling. The FSSH calculations were done with Newton‐X CS interfaced to ORCA.

## 3 Results and Discussion

### 3.1 Static Results

Prior to investigating the dynamics of the DPP dimers, it is helpful to discuss the electronic properties of the isolated monomer. Thus, we first analyze the low‐lying excited states of the DPP core and compare them to the DPP dimer.

**FIGURE 3:**
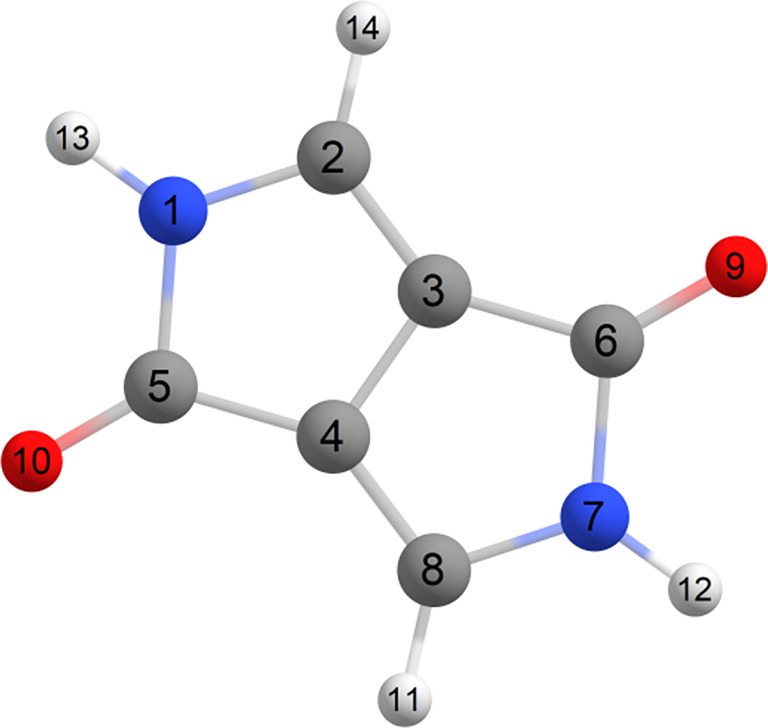
Molecular structure of DPP monomer.

The core of the DPP molecule (i.e., without functionalization) is shown in Figure 3. The optimization of the ground state returns a planar geometry. The normal modes were calculated to confirm that it is a minimum; the IR spectrum is given in Supporting Information S2.

Vertical excitation energies of DPP using TDDFT (CAM‐B3LYP/def2‐TZVP+D4) are given in Table 1 (CC2 results are shown in Supporting Information S1). It can be noticed that the excitations are either $\pi \to \pi^{*}$ or $n_{O}\to \pi^{*}$ transitions. The first excited state is the brightest transition. The corresponding NTOs for these transitions are also shown in Supporting Information S2.

**TABLE 1:** DPP‐monomer state character, vertical excitation energy, and oscillator strength calculated with TDDFT/CAM‐B3LYP/def2‐TZVP+D4 for the first six excited singlet states.

State	Transition	$E_{\mathrm{exc}}\;\left[\mathrm{eV}\right]$	$f_{\mathrm{osc}}$
S_1_	$\pi \to \pi^{*}$	3.56	0.257
S_2_	$n_{O}\to \pi^{*}$	4.12	0.000
S_3_	$\pi \to \pi^{*}$	4.12	0.000
S_4_	$n_{O}\to \pi^{*}$	4.58	0.000
S_5_	$\pi \to \pi^{*}$	5.52	0.000
S_6_	$\pi \to \pi^{*}$	6.51	0.000

The minimum energy geometry of the DPP dimer was next explored, a complex task due to the numerous possible molecular orientations. Various initial guess structures, including sandwich, T‐shaped, and parallel‐displaced configurations, were examined. Following optimization at the B3LYP/def2‐TZVP+D4 level, all guesses converged to one of the three dimers shown in Supporting Information S3 (their Cartesian coordinates are also given in the Supporting Information S1–S9). The CC2 results corroborate the DFT findings. Based on the Boltzmann distribution of DFT ground‐state energies (Table S7), Dimer 2's population at 300 K is predicted to be only 23% of Dimer 1's, with Dimer 3 making a negligible contribution at this temperature. While Dimer 2's existence is not negligible, the high computational cost of the dynamics prevents us from conducting surface hopping simulations for it. Therefore, we limit our analysis to dynamics originating from Dimer 1.

**FIGURE 4:**
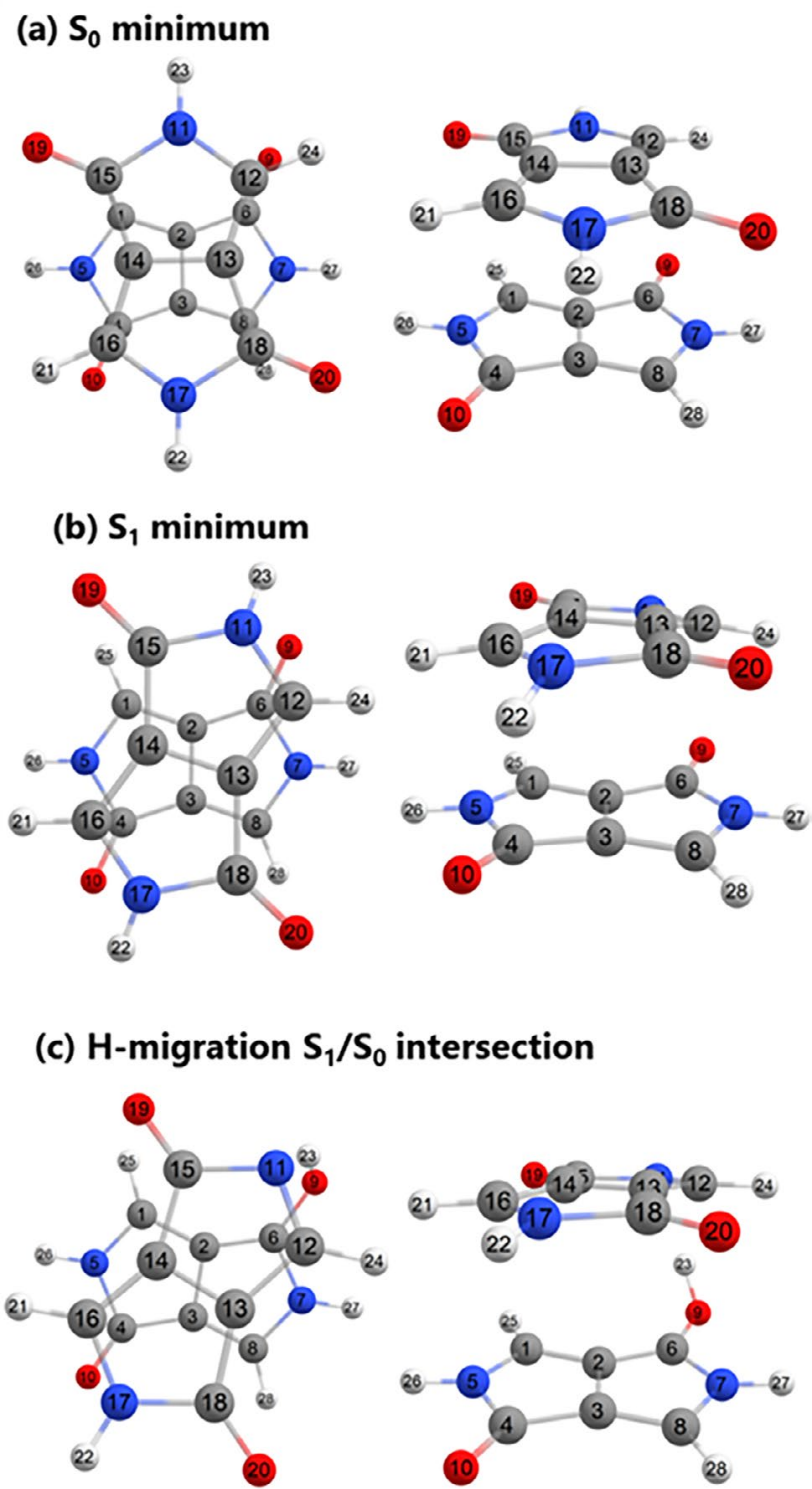
Optimized structures of S_0_ (a), S_1_ (b), and S_1_/S_0_ intersection (c) of the DPP‐dimer (frontal and side view) and atoms numbering.

The lowest‐energy S_0_‐optimized dimer (Dimer 1) is shown in Figure 4a. Two things can be noticed here. First, the two monomers are not entirely planar anymore due to the interaction between the oxygens in one monomer and the hydrogens of the pyrrolo groups in the other monomer; the second observation is that the dimer does not present typical π‐stacking geometries but an almost perfect perpendicular face‐to‐face orientation, which makes the two molecules decoupled. This can be seen in Table 2 as the excitation energies are degenerated in pairs. Nonetheless, the two chromophores are held together by noncovalent interactions, which is manifested in the total electronic energy of the dimer, which is 0.63 eV smaller than twice the energy of the monomer.

**TABLE 2:** DPP‐dimer state character, vertical excitation energy, oscillator strength, and absorption wavelength calculated with TDDFT/CAM‐B3LYP/def2‐TZVP+D4 for the first six excited singlet states.

State	Transition	$E_{\mathrm{exc}}\;\left[\mathrm{eV}\right]$	$f_{\mathrm{osc}}$
S_1_	$\pi \to \pi^{*}$	3.41	0.111
S_2_	$\pi \to \pi^{*}$	3.41	0.111
S_3_	$\pi \to \pi^{*}$	3.70	0.072
S_4_	$\pi \to \pi^{*}$	3.70	0.072
S_5_	$\pi \to \pi^{*}$	4.05	0.000
S_6_	$\pi \to \pi^{*}$	4.07	0.000

**FIGURE 5:**
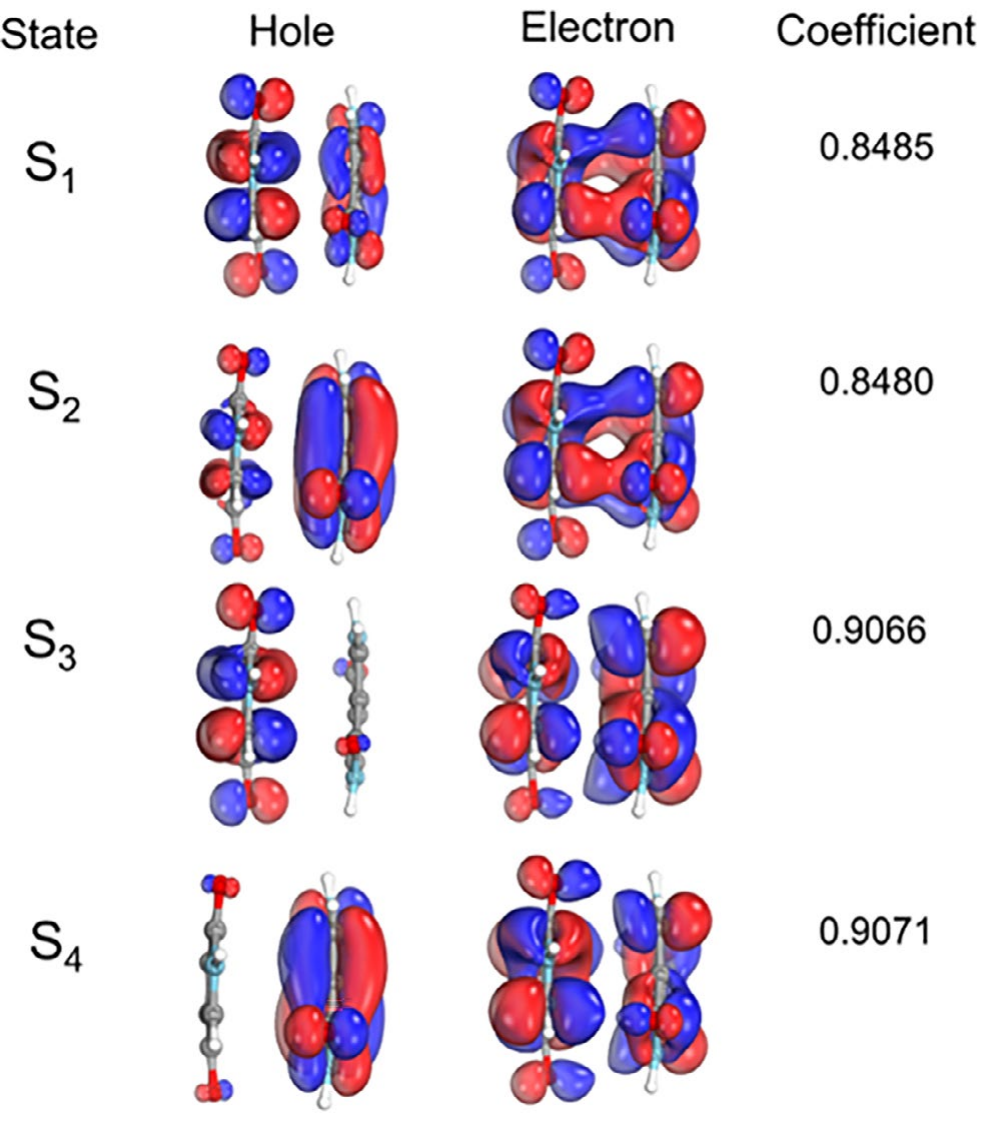
NTOs for the DPP dimer at the S_0_ minimum (level TDDFT/CAM‐B3LYP/def2‐TZVP+D4).

Table 2 presents the vertical excitation energies computed at the TDDFT level. These calculations were repeated with DFT/MRCI, ADC(2), and ODM3/MRCI and are shown in Supporting Information S1. These comparisons confirm that TDDFT/CAM‐B3LYP/def2‐TZVP+D4 can adequately describe the DPP dimer. Table 2 presents that the first excited state of the dimer is 0.15 eV below the monomer (3.41 vs. 3.56 eV). The NTOs of the dimer (Figure 5) show that the first four excited states have electronic transitions delocalized into the two chromophores, although S_3_ and S_4_ have the HOMO contribution in only one monomer.

**FIGURE 6:**
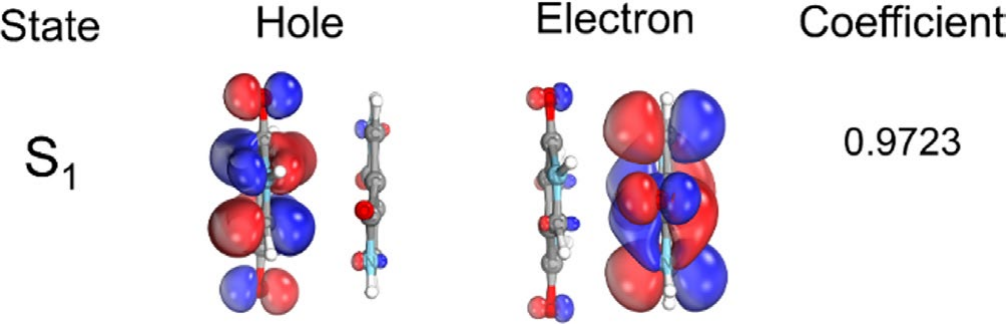
NTOs for the DPP dimer at the S_1_ minimum (TDDFT/CAM‐B3LYP/def2‐TZVP+D4).

The S_1_ optimized geometry is characterized mainly by a change in the improper dihedral angle between the chromophores (atoms 5–7 to 11‐17) from 72° in S_0_ minimum to about 57° in S_1_ minimum. This change corresponds to a relative rotation between the two molecules, which favors the N—H—O interaction in the first excited state, as can be observed by comparing Figure 4a,b. The S_1_ minimum geometry is confirmed by the absence of imaginary frequencies, as shown in Supporting Information S4. The NTOs at the S_1_ minimum are shown in Figure 6. The hole and electron are localized in different monomers, indicating the formation of a CT state or a CT exciton.

The molecular relaxation from the vertical excitation region to the first excited state is more efficient in the DPP dimer compared with the monomer (0.49 compared with 0.16 eV). This means that the dimer has more kinetic energy (compared with the monomer) to overcome energy barriers (transition states) in the S_1_ state.

### 3.2 Dynamics

FSSH was performed with TDA approximation, a simplification of TDDFT, which neglects the off‐diagonal coupling term between excitations and de‐excitations. This approximation makes TDA more affordable for running nonadiabatic dynamics. Therefore, before discussing the surface hopping results, we show how TDA/CAM‐B3LYP/6‐31G** + D4 compares with those at TDDFT/CAM‐B3LYP/def2‐TZVP+D4.

Table 3 presents the vertical excitation energies compared with both levels of theory. These results show some noticeable differences between the two computational levels. The excitation energies are ~0.13 eV apart, which is within the error bar of the method. The oscillator strengths, however, are different by a factor of one third. Such a difference is expected, as it is well known that oscillator strengths are not well predicted within TDA approximation [22]. Fortunately, this difference does not impact the dynamics because nonadiabatic couplings computed with TDBA use only information on energy and its second derivative and do not depend on state character (as oscillator strengths do). Moreover, Hu et al. have argued that TDA can even perform better than TDDFT near intersections between states due to partial compensation for local density approximation errors [22]. Thus, we safely adopted TDA to perform the surface hopping dynamics simulations.

**TABLE 3:** Comparison between the vertical excitation energies and oscillator strengths computed for the DPP dimer with different methods.

Methods	TDDFT/def2‐TZVP+D4		TDA/6‐31G** + D4	
State	*E*_exc_ [eV]	*f*_osc_	*E*_exc_ [eV]	*f*_osc_
S_1_	3.41	0.111	3.53	0.033
S_2_	3.41	0.111	3.53	0.034

**FIGURE 7:**
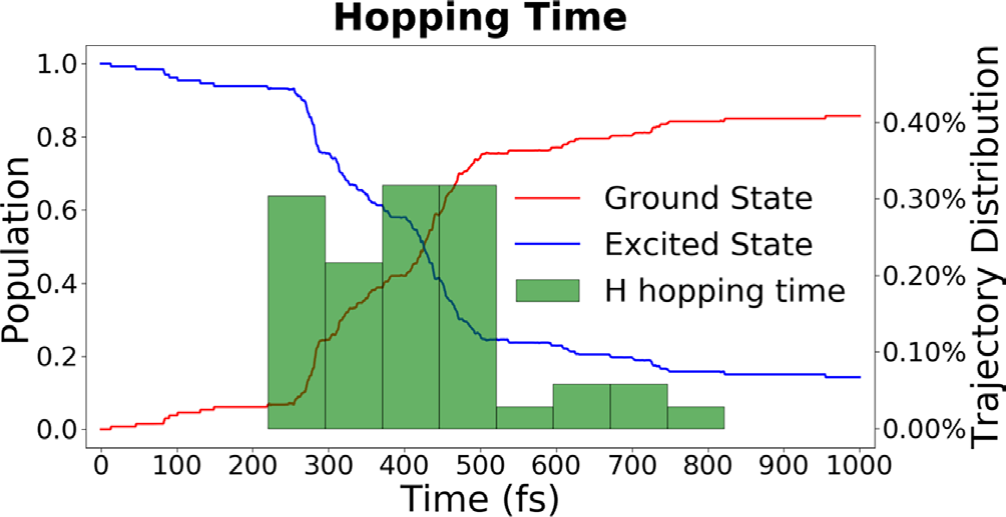
Ground and excited‐state population evolution of the DPP dimer. The excited‐state population is the sum of S_2_ and S_1_ populations. The histogram shows the distribution of S_1_ to S_0_ hoppings due to the H‐migration process.

The ground‐ and excited‐state population of DPP dimer in the first picosecond after photoexcitation are shown in Figure 7. The excited‐state population is the sum of the population of both the first and second excited states. Complete relaxation from S_2_ to S_1_ occurs within only 20 fs, as shown in Supporting Information S5. The population of a state at each time step is calculated as the sum of all trajectory populations of that state normalized to the number of trajectories.

We can see in Figure 7 that the excited‐state population quickly drops due to internal conversion. Such a short lifetime indicates that this DPP dimer is not fluorescent, which is somewhat surprising since DPP derivatives and DPP crystals are reported to be strongly fluorescent in the literature [25, 26]. To examine the cause of such a short‐lived excited state in the DPP dimer, we should investigate the origin of the internal conversion during dynamics.

Table 4 presents the statistics for the 120 simulated trajectories in terms of hopping geometries. Only 8 (7%) trajectories were still in the excited states at the end of the dynamics. The hoppings to the ground state were divided into two types. The first is hydrogen migration from the amino group to the carbonyl oxygen atom in the other monomer (we will show in Section 3.3 that a hydrogen atom and not a proton is transferred between monomers). The H‐migration leads to an S_1_/S_0_ intersection, with hopping geometries having almost zero S_1_—S_0_ energy gap (Δ*E*). It is clearly the dominant relaxation process of 92 trajectories (77%). As a remark, we should bear in mind that, at the TDDFT level, the branching space surrounding the intersection between S_0_ and S_1_ state has a dimensionality of one, not two [27]. Therefore, those intersection points are not rigorously conical at this theoretical level.

**TABLE 4:** Classification of the trajectories in terms of hopping geometries.

Type of trajectory	Counts	%
Survived in S_1_	8	7 ± 4
H‐migration hops (Δ*E* ≈ 0 eV)	92	77 ± 8
Weak coupling hops (Δ*E* > 1 eV)	20	16 ± 7

*Note:* Δ*E* is the S_1_—S_0_ energy gap. Error bars were estimated for a 95% confidence interval.

Second, we observed what we call weak coupling hoppings, characterized by occurring at an energy gap of more than 1 eV. These hoppings made up 20 trajectories (16%). Most of these trajectories did not show any eye‐catching geometric distortion. One of them had a CH—O inter‐monomers interaction, and another one featured a CH bond breaking. The weak coupling hoppings arise from the stochastic nature of the surface hopping process, which happens if the randomly generated number is smaller than the hopping probability at that specific time step. If the number of time steps is large enough, even tiny probabilities associated with large energy gaps can yield hopping events [28]. We verified that this was the case for the weak coupling hoppings. Because the primary results in this paper are the internal conversion due to H‐migration, we did not investigate further if the weak couplings were actual nonadiabatic events or an artifact of the computational level. We note, however, that they have been reported in other case studies, too [29, 30].

The population decay that is shown in Figure 7 is composed of both processes, H‐migration (which dominates the dynamics) and weak coupling hoppings. The weak coupling hoppings are spread throughout the trajectories, as expected from an exponential decay pattern. Nevertheless, the H‐migration hoppings require some time to be triggered. For this reason, either a single exponential or a multiexponential decay function does not correctly fit the population decay. The H‐migration hopping only occurs after an exited‐state intermediate is formed, implying that a sigmoid function should describe it. Sigmoid decay is not usually used to describe the decay of photophysical processes. Still, there are examples in the literature that have similar observations, such as for coumaryl Meldrum and sinapoyl Meldrum [31] and other systems [32, 33]. The sigmoid function is given by:

$$p\left(t\right)=\frac{a}{1+e^{\frac{t-\tau_{L}}{\tau_{E}}}}$$

and its fit is shown in Supporting Information S5.

The time constant $\tau_{L}$ was found to be 348 fs, which is the time at which half of the population is relaxed to the ground state. During the H‐migration intersection, the population decay follows the time constant of $\tau_{E}$. Before decaying to the ground state, an excited state minimum is formed at the time $\tau_{L}-\tau_{E}$, which means around 300 fs. Thus, the internal conversion lifetime $\tau_{\mathrm{IC}}$ at which the population decay is decreased by a factor of a 1/e can be calculated as

$$\tau_{\mathrm{IC}}=\tau_{E}\ln\left(e-1\right)+\tau_{L}$$

Thus, the internal conversion lifetime is 427 fs.

The geometry corresponding to the H‐migration intersection is shown in Figure 4c. It can be observed that the geometry is very similar to the S_1_ minimum in terms of the orientation angle between the chromophores and differs in terms of O—H bond length. This means that the 300‐fs required to trigger the relaxation pathway will include the time to relax from the S_0_ to S_1_ minima.

**FIGURE 8:**
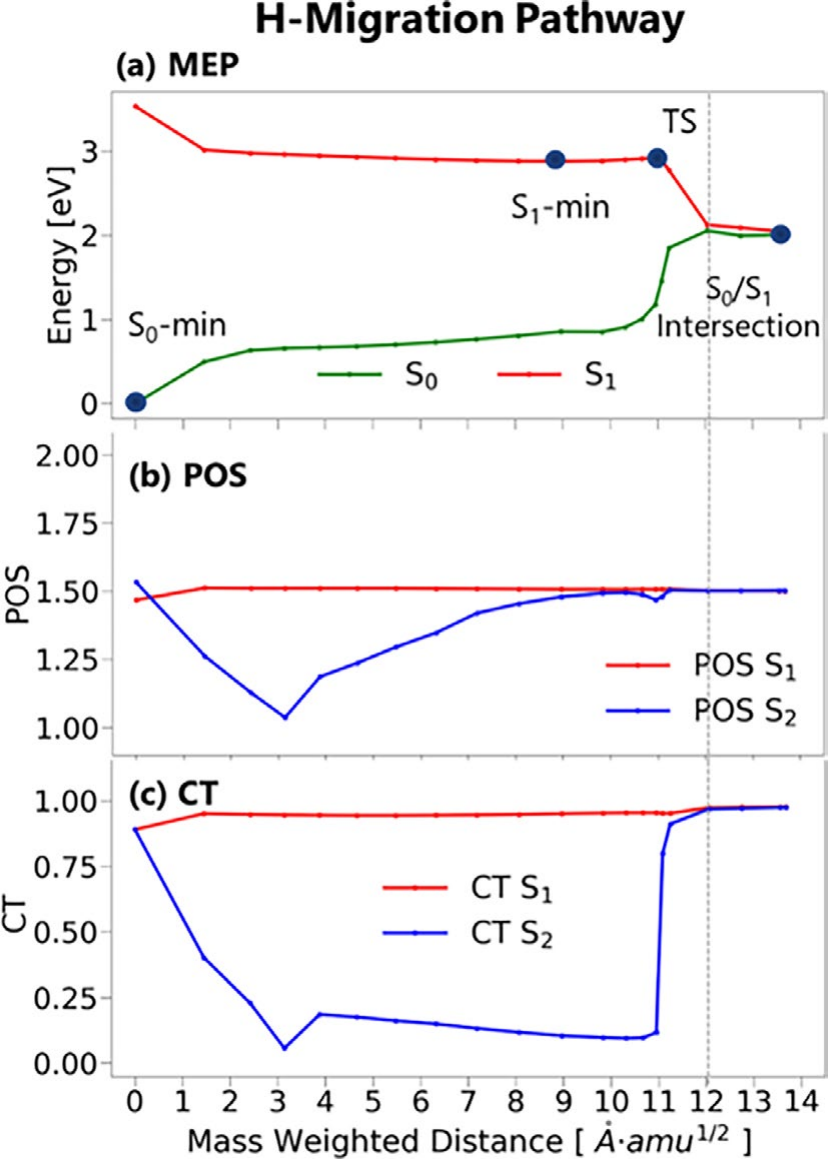
Potential energy profile (NEB) of (a) minimum energy path (MEP), (b) POS, and (c) CT connecting the S_0_ minimum, S_1_ minimum, S_1_ transition state, and S_1_/S_0_ H‐migration intersection as a function of the mass‐weighted distance to the S_0_ minimum. Calculated with TDA/CAM‐B3LYP/6‐31G** + D4. The vertical line indicates the point where the fragment definition changes.

To understand how accessible the H‐migration intersection relaxation pathway is, we investigated its potential energy profile, plotted in Figure 8a. This pathway was calculated using the NEB method to find the minimum energy pathway between the S_0_ minimum and S_1_ minimum and between the S_1_ minimum and the S_1_/S_0_ H‐migration intersection. The transition state was optimized and confirmed to have a single imaginary frequency corresponding to the hydrogen migration. Mass‐weighted distances were used to quantify the degree of differences between the initial structure and the corresponding intermediates.

From the S_1_ minimum, we found an activation barrier of 0.04 eV, which is much smaller than the kinetic energy excess coming from the relaxation after excitation at the Franck‐Condon region (0.65 eV). The same energy profile was constructed with the bigger def2‐TZVP basis set, and a similar result was obtained (see Supporting Information S6). The pathway toward the S_1_ minimum formation requires a distortion of about 9 Å.amu^1/2^ from the S_0_ optimized geometry. After crossing the transition state, reaching the crossing seam requires only about 1 Å.amu^1/2^ distortion. The virtually barrierless H‐migration pathway should be easily overcome at room temperature, even if energy dissipation into a crystalline or solvent environment is considered (and supposing that this environment does not alter the barrier).

### 3.3 Electronic Transitions Characterization

We have characterized the electronic transitions along the pathway in terms of CT using the TheoDORE program. This program uses the one‐electron transition density matrix to calculate descriptors such as CT number and average POS [34]. The equations governing both are discussed in Supporting Information S7. The CT value ranges from zero to one, where zero indicates no CT, and one indicates one electron transfer. POS, on the other hand, varies from one to two. If the number is close to one or two, it suggests an intra‐excitation within the same monomer. In contrast, a value around 1.5 shows an inter‐excitation between the fragments or a transition with the hole and the electron delocalized in the whole dimer. Both descriptors require the definition of specific fragments involved in the electronic transitions. We defined these fragments as each one of the chromophores. After the transition state, we considered one fragment deprotonated and one additional hydrogen atom in the second fragment. Until now, the isomerization triggering internal conversion has been regarded as an H‐migration, even though we have not discussed whether it is a hydrogen or a proton transfer. The CT descriptor along the H‐migration pathway presented in Figure 8c allows resolving between the two processes. The S_1_ state has a strong CT character during the entire pathway. Therefore, the proton transfer balances the electron transfer. Since a proton and an electron are transferred, the process is a hydrogen migration. This type of transfer is well‐known, being a subset of proton‐coupled electron transfer (PCET) [35] and named electron‐driven proton transfer (EDPT) [36]. Conical intersections along such pathways have been characterized before in other systems [37, 38].

**FIGURE 9:**
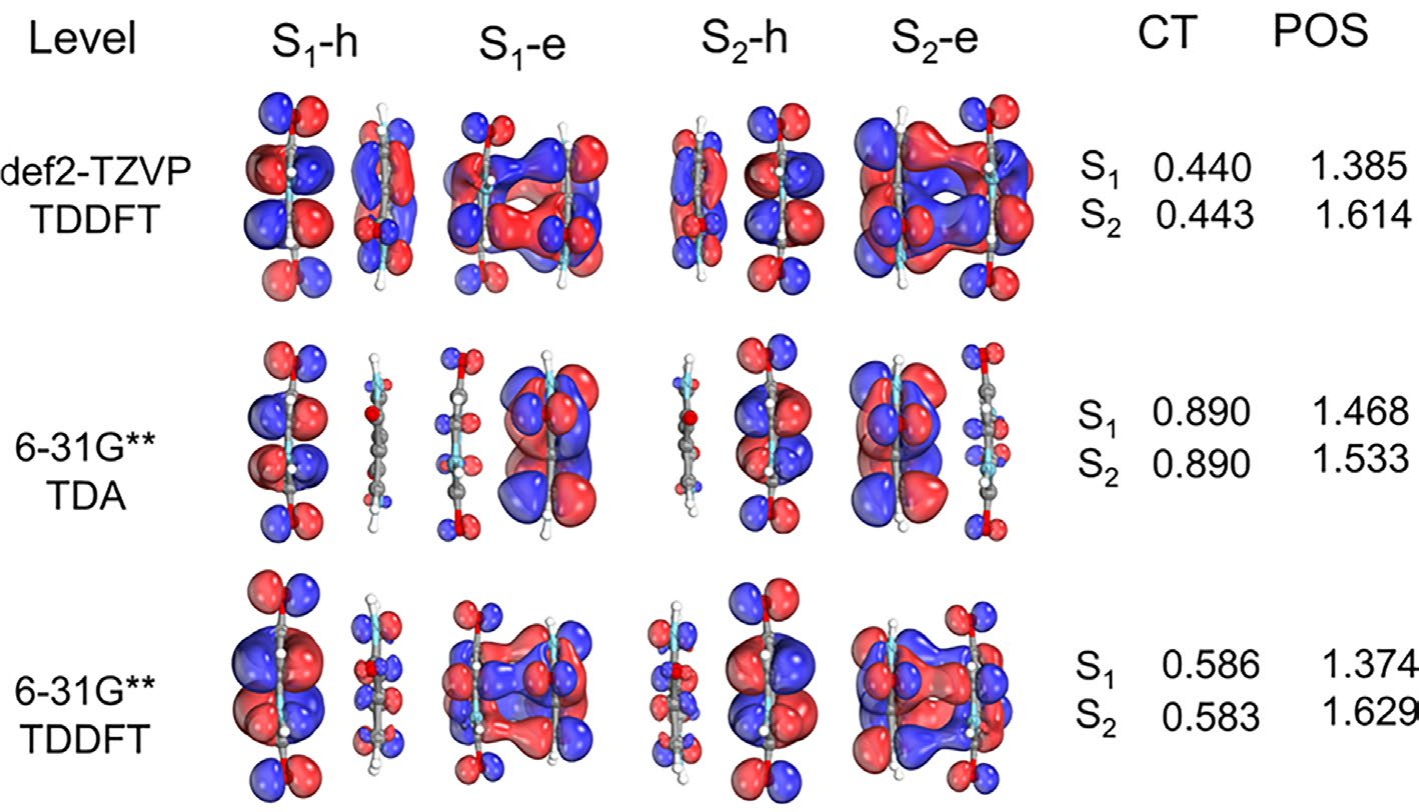
NTO, CT, and POS numbers are calculated for the S_1_ state at the S_0_ geometry with different approaches and basis sets.

We can observe that POS for S_1_ is constant along the pathway. This is because the first excited state is characterized by an intermolecular $\pi \to \pi^{*}$ transition between the chromophores. Hence, the average value is 1.5. This means that the average exciton position remains between the two fragments along the reaction coordinate, which makes sense, as the hole and electron are localized in different fragments along the whole pathway. Nonetheless, we see a tiny dip in the first point of the PES (S_0_ minimum), where the POS‐S_1_ slightly shifts to fragment one. This is observed because, in this case, the LUMO is not fully localized in one chromophore, like in the case shown in Figure 6. Thus, there is more contribution from one chromophore than the other (Figure 9). This dip in the S_0_ geometry is reversed in S_2_ since the states are degenerate.

We also noted that this dip exists in the CT‐S_1_, and it is frankly underestimated based on our static calculations. If we check the NTOs in Figure 5, we can see that the S_0_ → S_1_ transition is completely delocalized in the two chromophores; thus, a CT smaller than 0.5 would be expected, which indicates a local excitation (LE). However, as can be seen in Figure 8, this value is much larger (0.890).

When trying to understand those differences, we found some interesting results. We first checked if this large CT value at the S_0_ minimum would be related to the different computational levels used in the static calculations (TDDFT/CAM‐B3LYP/def2‐TZVP+D4) and in the dynamics and NEB calculations (TDA/CAM‐B3LYP/6‐31G** + D4). Even though the Kohn‐Sham orbitals are very similar in the two basis sets, their corresponding NTOs are different (Figure 9). In the first case, the hole and electron are entirely delocalized in the two fragments; in the other case, the hole and electron are localized in different fragments. This results in very different CT numbers (0.44 vs. 0.89). When we increased the basis set to def2‐TZVP, the problem persisted. However, when we turned off the TDA approximation, we got CT numbers consistent with our initial static calculations; that is, the S_1_ excitation has a localized character (LE). This means that setting **B** = 0 in the Casida equations [39] can alter the NTOs. This is because all contributions to the excitation energies coming from the de‐excitation of the correlated ground state are neglected.

This analysis implies that TDDFT results are more reliable when characterizing CT numbers and electronic transitions. However, this error does not propagate during the dynamics, as it occurs exclusively near the S_0_ minimum geometry. The differences between TDA and TDDFT dissipate rapidly as the system moves away from the Franck‐Condon region. Using the CT descriptor in S_1_ as a key metric for characterizing the state, the discrepancy observed at the S_0_ minimum (0.89 in TDA vs. 0.59 in TDDFT) persists in the initial conditions of the dynamics. For instance, in TRAJ1, a representative trajectory involving H‐transfer, S_1_ CT at time zero, is 0.61 for TDA compared with 0.32 for TDDFT. However, after the shearing motion that positions the dimer at the S_1_ minimum—prior to proton transfer—this value reaches 0.93 with TDA and 0.90 with TDDFT (calculated at 200 fs for TRAJ1). Thus, we are confident that TDA accurately captures the time evolution of the excited states during DPP dimer dynamics.

## 4 Conclusions

In this work, we have investigated the ultrafast excited‐state dynamics of diketopyrrolopyrrole (DPP) stacked dimers, which are promising materials for organic photovoltaic and semiconductor applications. Contrary to the expected strong fluorescence behavior typical of DPP derivatives, our NAMD simulations revealed a rapid internal conversion pathway driven by a near‐barrierless hydrogen migration. This process, occurring at an S_1_/S_0_ intersection, led to a short excited‐state lifetime of approximately 427 fs, which we attribute to a hydrogen atom transfer mechanism. This rapid internal conversion limits the photophysical performance of nonfunctionalized DPP stacked dimers, highlighting a critical challenge for their application in optoelectronic devices where prolonged excited‐state lifetimes are crucial for charge transport and energy harvesting.

Our findings demonstrate that hydrogen migration dominates nonradiative decay, being responsible for more than 75% of the relaxation to the ground state. The population decay of the excited state was observed to follow a sigmoid model. Internal conversion is only triggered after relaxation from the initially excited S_0_ to the S_1_ minima, which takes about 300 fs. The decay to the ground state is triggered by the transfer of hydrogen between the two chromophores, which brings the dimmer close to an S_1_/S_0_ intersection.

Thus, we conclude that the functionalization of DPP to block hydrogen migration is crucial to avoid internal conversion at early times and sustain favorable photophysical properties for its use in OPVs. In Supporting Information S8, we show that this ultrafast decay pathway could be mostly suppressed when replacing the hydrogens in the pyrrolo groups with methyl groups.

These results provide insights into the rational design of DPP‐based materials, where molecular functionalization can be used to manipulate excited‐state dynamics and improve the efficiency of organic semiconductors. Future work should focus on exploring various functionalizations to inhibit nonradiative decay and promote longer excited‐state lifetimes, opening new possibilities for enhancing the performance of DPP‐based optoelectronic devices.

## Author Contributions

Conceptualization: A.A.‐J, J.M.T., and M.B. Funding acquisition: M.B. Investigation: A.A.‐J. Methodology: A.A.‐J., J.M.T., and M.B. Project administration: M.B. Supervision: J.M.T., M.B. Visualization: A.A.‐J. Writing – original draft: A.A.‐J. Writing – review and editing: A.A.‐J., J.M.T., and M.B.

## Supporting information


**Data S1** Supporting Information.

## Data Availability

The data that support the findings of this study are available in the Supporting Information S1–S9 of this article. Molecular geometries and dynamics are available at 10.5281/zenodo.13915309.
