# Supplementary material for: Ultrafast Dynamics of Diketopyrrolopyrrole Dimers
Source: J Comput Chem. 2024 Dec 14;46(1):e27547. doi: 10.1002/jcc.27547 (PMC11645985; doi:10.1002/jcc.27547)
Supplement: Supplementary file 1 — Data S1 Supporting Information. [file JCC-46-0-s001.pdf]

Supplementary Material

Ultrafast Dynamics of Diketopyrrolopyrrole (DPP) Dimers

Ali Al-Jaaidi[1] | Josene M. Toldo[2] | Mario Barbatti[3]

[1] Aix Marseille University, CNRS, ICR, Marseille, France.
[2] Aix Marseille University, CNRS, ICR, Marseille, France.
UCBL, ENS de Lyon, CNRS, LCH, UMR 5182, 69342, Lyon cedex 07, France.
[3] Aix Marseille University, CNRS, ICR, Marseille, France.
Institut Universitaire de France, Paris, France.

Table of Contents

SM-1. ELECTRONIC STRUCTURE BENCHMARK 2
SM-2. GROUND-STATE CHARACTERIZATION 3
SM-3. DIMMER OPTIMIZATION 5
SM-4. S_1 MINIMUM CHARACTERIZATION 6
SM-5. POPULATION ANALYSIS 7
SM-6. H-MIGRATION PATHWAY 8
SM-7. DENSITY CHARACTERIZATION 8
SM-8. DYNAMICS OF SUBSTITUTED ME-DPP 9
SM-9. CARTESIAN COORDINATES 11

SM-1. ELECTRONIC STRUCTURE BENCHMARK

Table S-1: DPP dimer vertical excitation energies at different levels of theory.

State	Transition	E_{exc} $[eV]$	f_{osc}	λ $[nm]$	Orbitals
**ADC(2)/def2-TZVP**					
**S_1**	$\pi \rightarrow \pi^*$	3.09	0.178	401.5	70->71
**S_2**	$\pi \rightarrow \pi^*$	3.09	0.179	401.5	69->71
**S_3**	$\pi \rightarrow \pi^*$	3.53	0.025	351.3	70->72
**S_4**	$\pi \rightarrow \pi^*$	3.53	0.025	351.3	69->72
**S_5**	$n_O/\pi \rightarrow \pi^*$	3.55	0.000	349.2	65,66->71,72
**S_6**	$\pi \rightarrow \pi^*$	3.74	0.000	331.4	67,68->71,72
**ODM3/MRCI (12/10)**					
**S_1**	$\pi \rightarrow \pi^*$	3.86	0.331	320.9	50,49->51
**S_2**	$\pi \rightarrow \pi^*$	3.87	0.334	320.6	49,50->51
**S_3**	$\pi \rightarrow \pi^*$	4.13	0.000	300.4	48,47->51,52
**S_4**	$\pi \rightarrow \pi^*$	4.15	0.000	298.8	47,48->51,52
**S_5**	$\pi \rightarrow \pi^*$	4.24	0.000	292.4	49,50->51 D
**S_6**	$\pi \rightarrow \pi^*$	4.39	0.033	282.3	50->52
**TD-DFT/CAM-B3LYP/def2-TZVP**					
**S_1**	$\pi \rightarrow \pi^*$	3.41	0.111	363.3	70->71
**S_2**	$\pi \rightarrow \pi^*$	3.41	0.111	363.2	69->71
**S_3**	$\pi \rightarrow \pi^*$	3.70	0.072	334.9	70->72
**S_4**	$\pi \rightarrow \pi^*$	3.70	0.072	334.8	69->72
**S_5**	$\pi \rightarrow \pi^*$	4.05	0.000	306.3	68,66->71,72
**S_6**	$\pi \rightarrow \pi^*$	4.07	0.000	304.8	68,66->72,71
**DFT/MRCI geometry optimized with DFT/B3LYP/def2-TZVP**					
**S_1**	$\pi \rightarrow \pi^*$	3.24	0.212	383	70->71
**S_2**	$\pi \rightarrow \pi^*$	3.24	0.213	383	69->71
**S_3**	$\pi \rightarrow \pi^*$	3.52	0.056	352	70->72
**S_4**	$\pi \rightarrow \pi^*$	3.52	0.055	352	69->72
**S_5**	$\pi \rightarrow \pi^*$	3.76	0.001	330	66,65->71,72
**S_6**	$\pi \rightarrow \pi^*$	3.76	0.000	330	65,66->71,72
**S_9**	$\pi \rightarrow \pi^*$	3.86	0.000	321	69,70->71

SM-2. GROUND-STATE CHARACTERIZATION

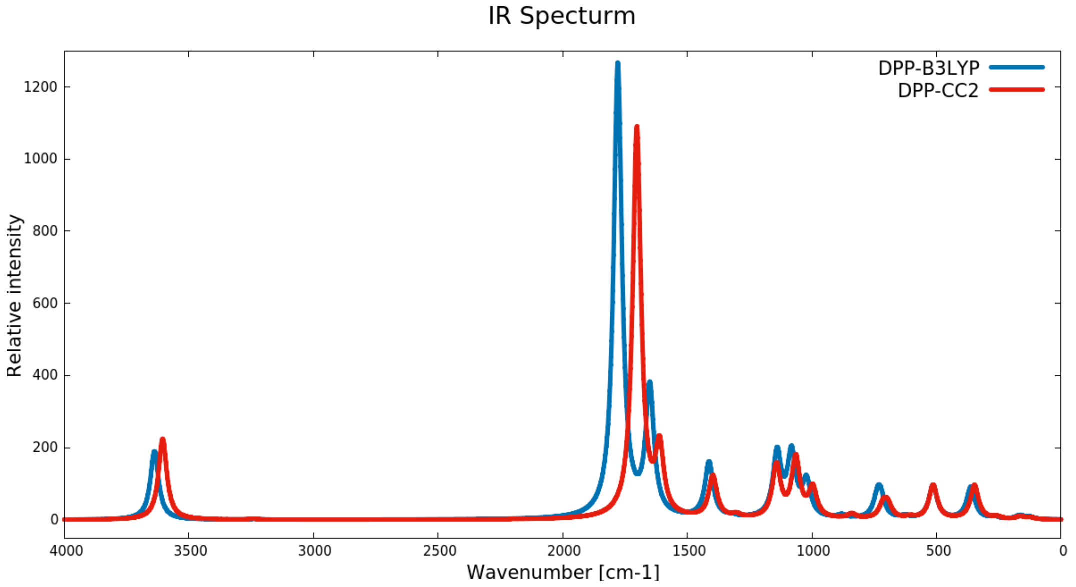

Figure S-2: DPP-Monomer IR Spectrum with DFT-B3LYP/def2-TZVP (in red) CC2/def2-TZVP (in blue) at the S_0 minimum.

State	Transition	E_{exc} $[eV]$	f_{osc}	λ $[nm]$
S_1	$\pi \rightarrow \pi^*$	3.45	0.298	359.3
S_2	$n_O \rightarrow \pi^*$	3.79	0.000	327.0
S_3	$\pi \rightarrow \pi^*$	3.89	0.000	319.0
S_4	$n_O \rightarrow \pi^*$	4.25	0.000	291.5
S_5	$\pi \rightarrow \pi^*$	5.28	0.000	234.8
S_6	$n_O \rightarrow \pi^*$	6.17	0.000	201.0

Table S-3: DPP-monomer state character, vertical excitation energy, oscillator strength, and absorption wavelength calculated with CC2/def2-TZVP for the first six excited singlet states.

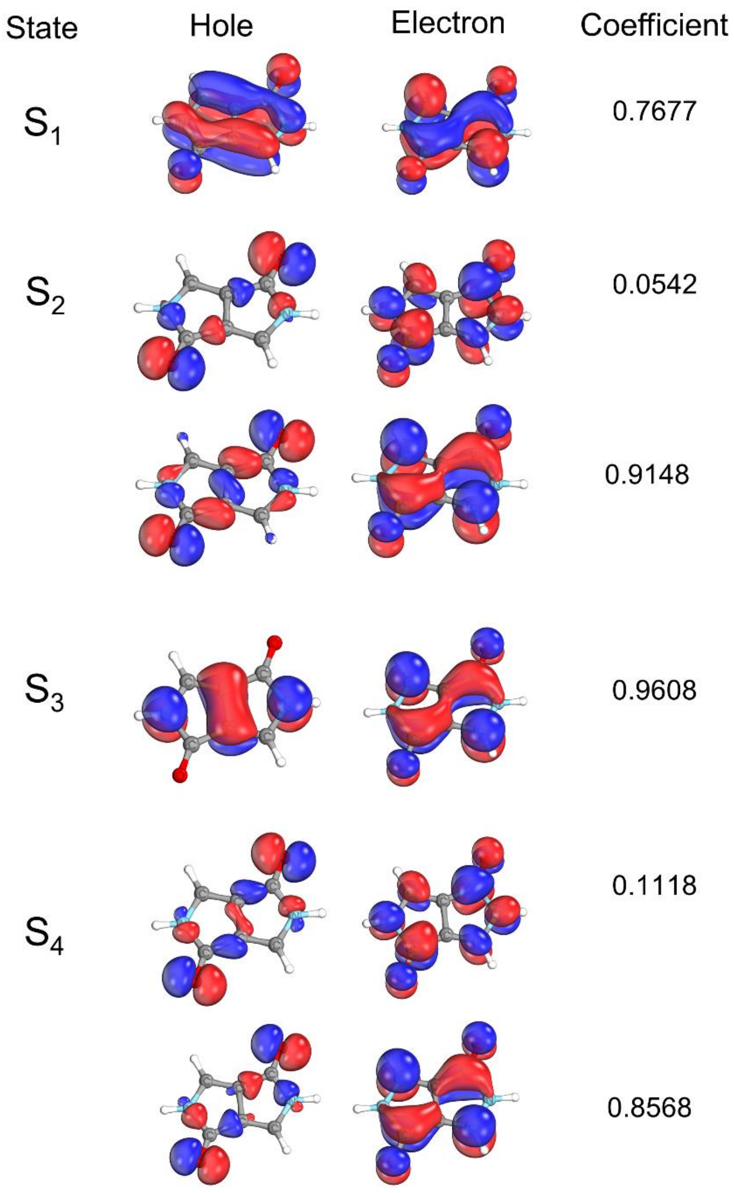

Figure S-4: NTOs for the first 4 electronic transitions for the DPP monomer at the S_0 minimum (level TDDFT/CAM-B3LYP/def2-TZVP).

SM-3. DIMMER OPTIMIZATION

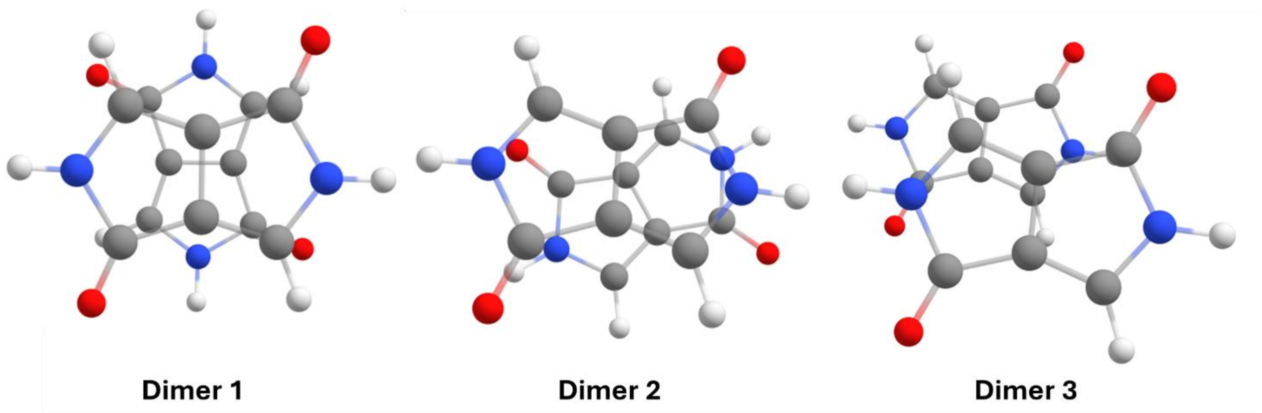

Figure S-5: DPP dimer guess structures for DFT-B3LYP/def2-TZVP+D4 optimizations.

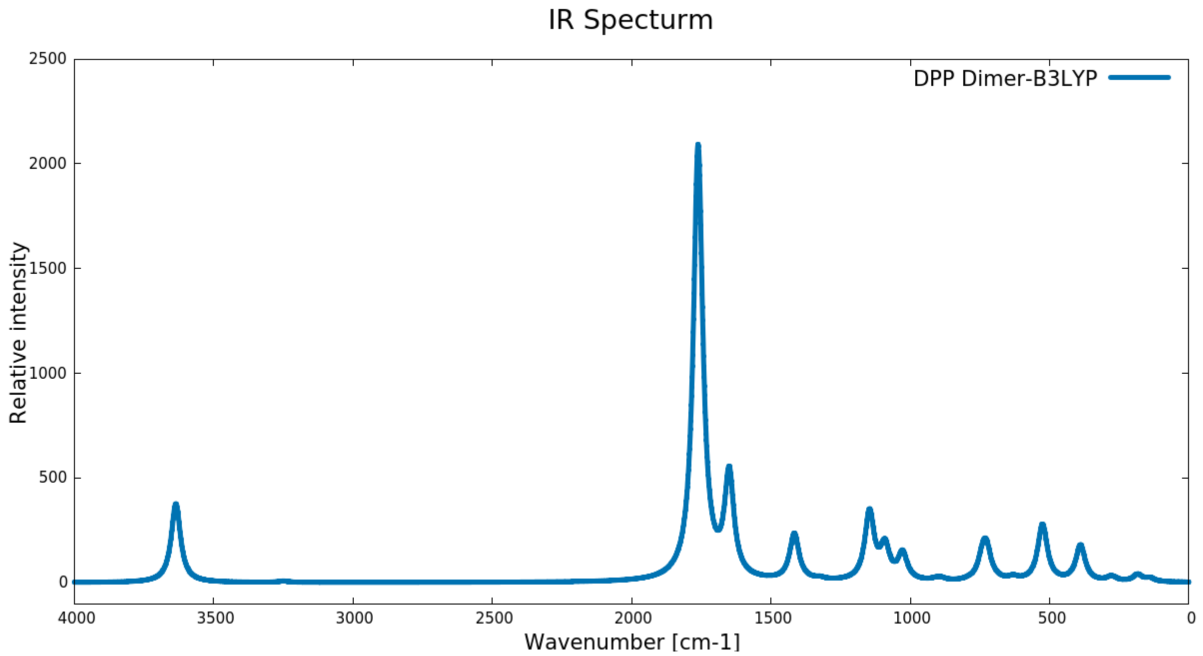

Figure S-6: DPP-Dimer IR Spectrum with DFT-B3LYP/def2-TZVP at the S_0 minimum.

Table S-7: Relative energies of the ground state minima found for different guess structures of DPP dimer at the DFT/B3LYP/def2-TZVP+D4 level.

Structure	DFT [eV]
**Dimer 1**	0
**Dimer 2**	0.038
**Dimer 3**	0.384

Table S-8: Relative energies of the ground state minima found for different guess structures of DPP dimer at the CC2/def2-TZVP level.

Structure	CC2 [eV]
**Dimer 1**	0
**Dimer 2**	0.085
**Dimer 3**	0.444

SM-4. S_1 MINIMUM CHARACTERIZATION

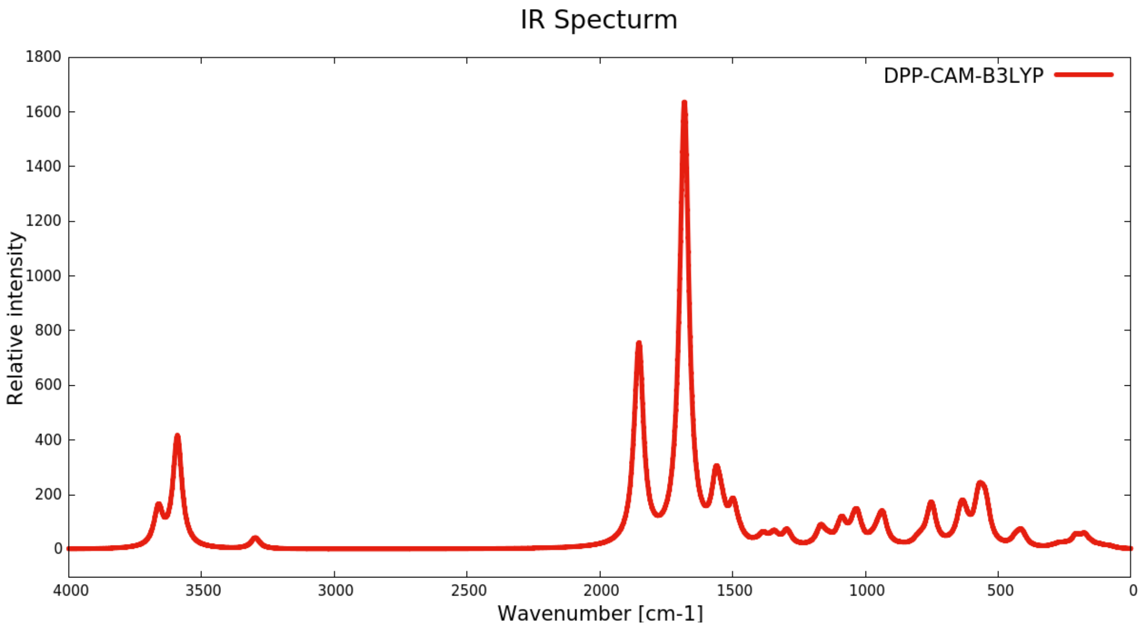

Figure S-9: DPP-Dimer IR Spectrum with TDDFT-CAM-B3LYP/def2-TZVP at the S_1 minimum.

Table S-10: DPP dimer S_1 vertical energies at S_0 and S_1 minimum at different levels of theory.

Method	S_1 [eV]	S_1 minimum [eV]
Monomer		
CC(2)/def2-TZVP	3.45	3.13
TD-DFT/CAM-B3LYP/def2-TZVP	3.56	3.21
ODM3/MRCI (5/3)	3.84	3.66
DFT/MRCI (TD-DFT optimized geometry)	3.39	3.08
Dimer (guess 2)		
ADC(2)/def2-TZVP	3.09	2.08
TD-DFT/CAM-B3LYP/def2-TZVP	3.41	2.26
ODM3/MRCI (5/3)	4.05	3.76
DFT/MRCI/def2-TZVP	3.24	2.20

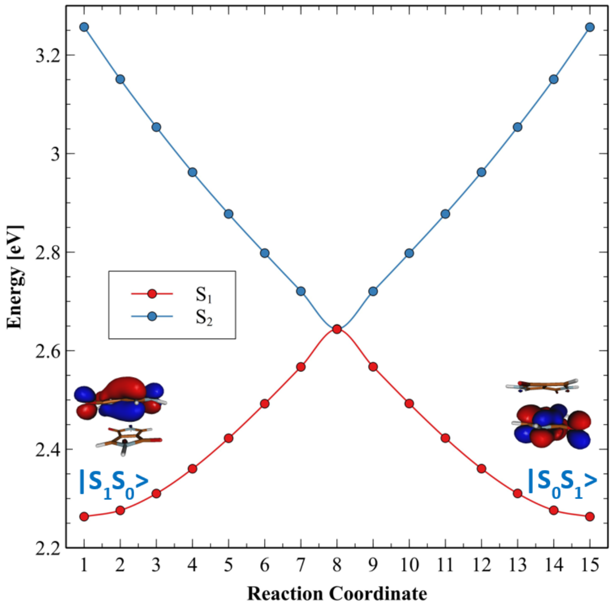

Figure S-11: Linear interpolation alongside the two degenerated S_1 minima for the DPP-dimer at TD-DFT/CAM-B3LYP/def2-TZVP.

SM-5. POPULATION ANALYSIS

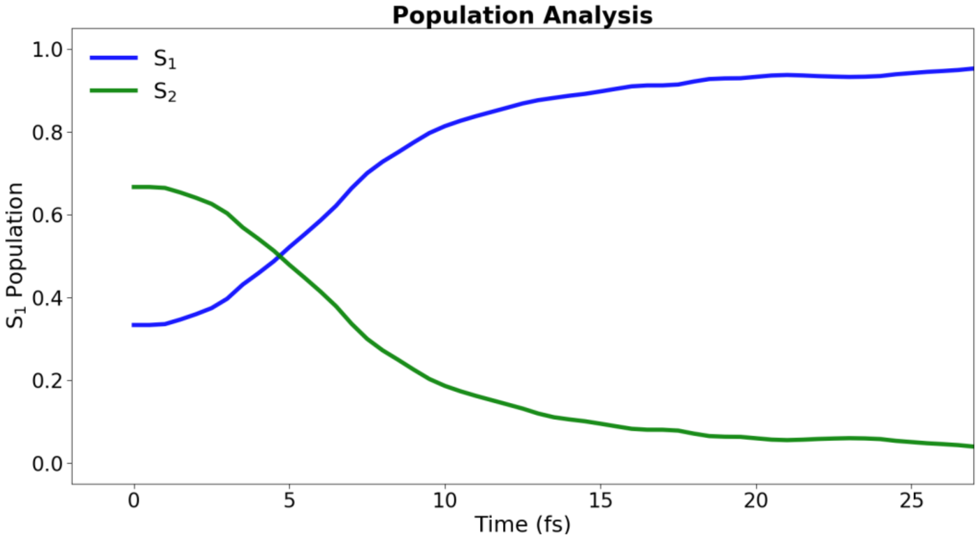

Figure S-12: Excited state population evolution (starting from the S_0). We can observe that immediate relaxation from S_2 to S_1 in about 25 fs.

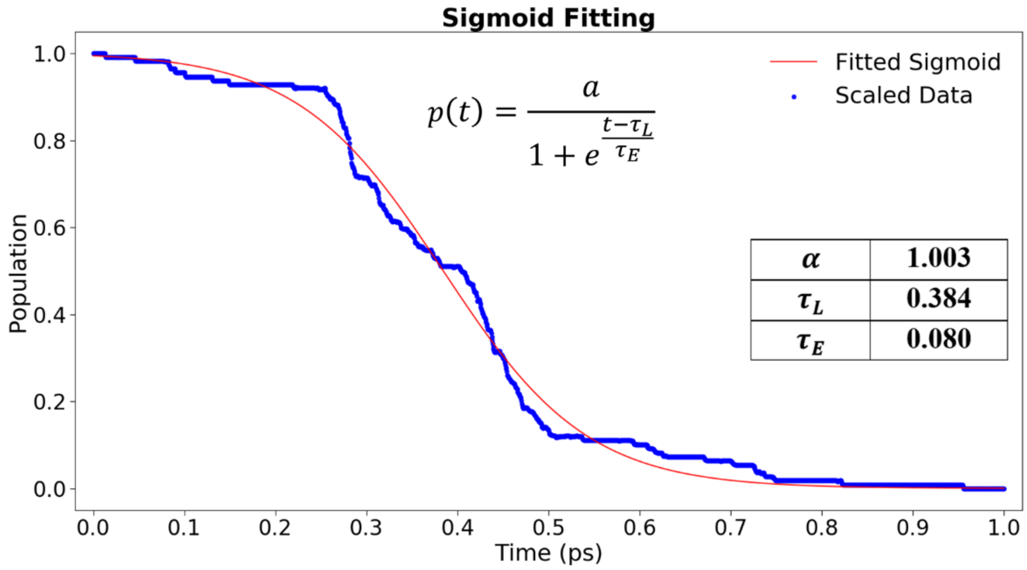

Figure S-13: Sigmoid fitting of the excited state population decay.

SM-6. H-MIGRATION PATHWAY

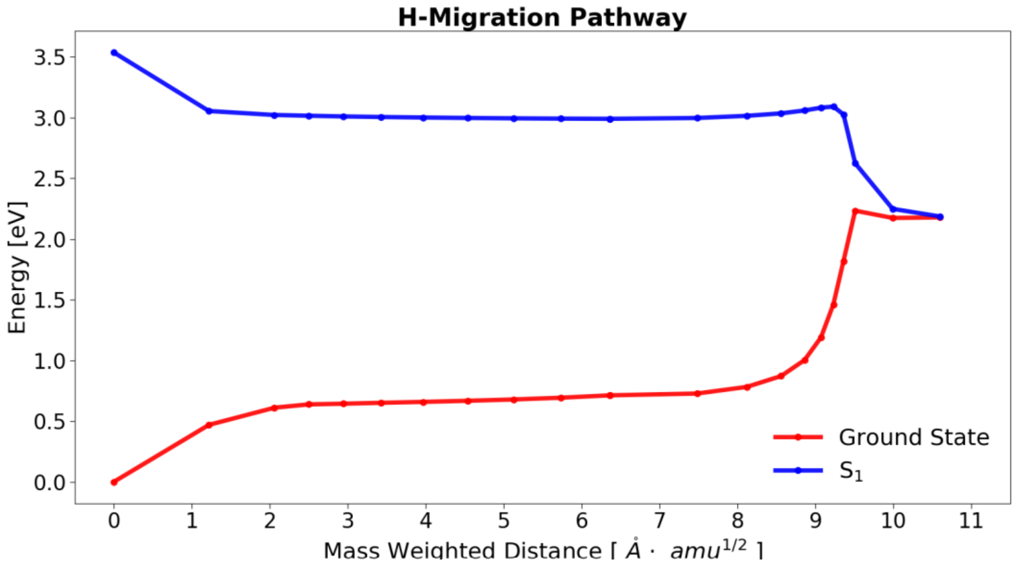

Figure S-14: Minimum energy path (MEP) connecting the S0 minimum, S1 minimum, S1 transition state, and S1/S0 H-migration intersection as a function of the mass-weighted distance to the S0 minimum. Calculated with TDA/CAM-B3LYP/def2-TZVP+D4.

SM-7. DENSITY CHARACTERIZATION

The characterization of excitation along the H migration pathway in DPP was done with TheoDORE program. Some of the relevant equations will be given here.

Consider a system consisting of multiple fragments. The localized orbital basis of the transition matrix element for a transition from the ground state to the *n*th excited state can be calculated as:

$$D_{ab}^{0n} = \langle 0|\hat{\mathcal{E}}_{ab}|\mathrm{n}\rangle \tag{1}$$

where $\hat{\mathcal{E}}_{ab}$ is an excitation operator for the orbitals a and b localized as fragments A and B, respectively. Thus, a charge transfer number Ω_{AB}^{n} for an excitation can be defined as:

$$\Omega_{AB}^{n} = \frac{1}{2}\sum_{\mu\in A}\sum_{v\in B}[\,(\boldsymbol{D^{0n}S})_{uv}(\boldsymbol{SD^{0n}})_{uv} + D_{uv}^{0n}(\boldsymbol{SD^{0n}S})_{uv}] \tag{2}$$

where *S* represents the orbital overlap matrix. This value indicates the contribution of charge transfer from fragment A to fragment B (when A≠B), and the contribution from excitations within the same fragment (when A=B). The total charge transfer character for a system with multiple fragments is defined as:

$$CT = \frac{1}{\Omega^{n}}\sum_{A}\sum_{B\neq A}\Omega_{AB}^{n} \tag{3}$$

with Ω^{n} representing the total sum of the charge transfer values for all pairs A and B.

We can also introduce the position of the excitation (POS) as:

$$POS = \frac{\sum_{A} A(\sum_{B}\Omega_{AB}^{n} + \Omega_{BA}^{n})}{2\Omega^{n}} \tag{4}$$

This quantity represents the localization of the transition. POS assumes values between 1 and 2. If POS = 1, it means that the transition is completely localized in chromophore 1 (hole and electron are in the same chromophore); if POS =2, the transition is completely localized in chromophore 2. Intermediary values imply a delocalized transition (either hole and electron localized in different chromophores or both, hole and electron, delocalized in the dimer).

SM-8. DYNAMICS OF SUBSTITUTED ME-DPP

Figure S-15 shows three sets of dynamics of the DPP dimer. The blue curve is the dynamics discussed in the paper, corresponding to initial conditions generated from a Wigner distribution in the S_0 minimum. The red curve shows the population when the initial condition sampling is done with a Wigner distribution in the S_1 minimum, aiming to emulate the dynamics after 0-0 excitation. The green curve shows the population when the DPP dimer is functionalized with methyl groups (Me-DPP), with initial conditions also sampled in the S_1 minimum. The two sets that start with the initial conditions sampled in the S_1 minimum used a full kinetic energy reservoir to evaluate back hoppings and had PESs computed at TDA/CAM-B3LYP/def2-SV(P)+D4 level. In contrast, dynamics sampled in the S_0 minimum used a reduced kinetic energy reservoir and was propagated with TDA/CAM-B3LYP/6-31G**+D4. Thus, the comparison between the first and the other two sets is not entirely consistent.

Comparison between the dynamics of the Me-DPP dimer (green curve in Figure S-15) and of DPP dimer (red curve), both sampled at S_1, shows how the methyl functionalization is efficient in suppressing the hydrogen conical intersection, which is evident as the substituent blocked this pathway. However, we still observe a decay to the ground state caused by the weak-coupling hoppings.

A comparison between the dynamics of the DPP dimer sampled in S_0 blue (blue curve in Figure S-15) and that of the DPP dimer sampled in S_1 (red curve) shows that when dynamics start near the S_1 minimum, internal conversion starts immediately. This confirms that the initial latency time after sampling S_0 is due to the relaxation to the S_1 minimum, as discussed in the paper.

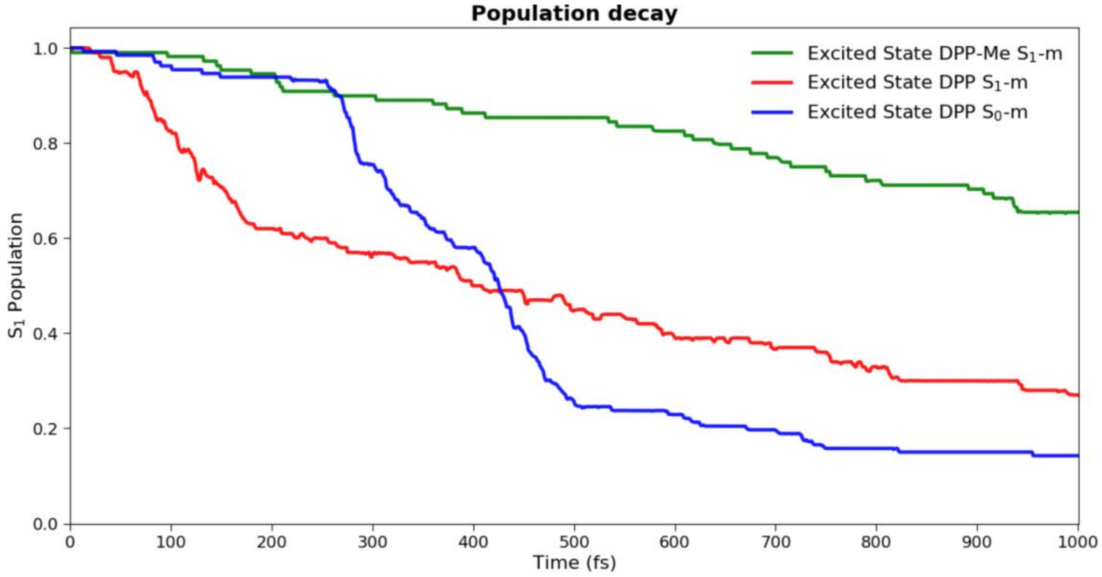

Figure S-15: Summary of the S_1 population decay for the three sets of dynamics. Red and blue curves are for DPP dimer and sampled from S_1 and S_0 minima, respectively. Green is the Me-DPP dimer sampled from S_1 minima.

SM-9. CARTESIAN COORDINATES

Cartesian coordinates (Å) of the dimers optimized with DFT/B3LYP/def2-TZVP+D4.

Dimer1

C	2.882772	2.720007	0.302811
C	1.576431	3.046186	0.488875
C	1.384827	3.349193	1.882284
C	2.652676	3.165725	2.573515
N	3.534107	2.816267	1.507441
C	0.280494	3.077810	-0.173223
N	-0.594531	3.461922	0.886190
C	0.061741	3.584938	2.085717
O	-0.087598	2.820769	-1.300609
O	2.983220	3.219887	3.739856
N	1.145409	-0.343479	-0.270460
C	-0.038881	-0.021556	0.344857
C	0.193574	0.142216	1.674014
C	1.596369	-0.070696	1.912679
C	2.243887	-0.353005	0.639921
C	1.845430	0.182919	3.224616
N	0.666094	0.531577	3.834843
C	-0.426050	0.575785	2.917840
O	3.399604	-0.520970	0.310229
O	-1.544426	0.945952	3.208878
H	2.770603	0.169287	3.778206
H	0.580708	0.851347	4.785508
H	1.272857	-0.434971	-1.264784
H	-0.946483	0.087107	-0.226991
H	3.406656	2.401717	-0.584237
H	4.490771	2.548123	1.669274
H	-1.593353	3.502733	0.767934
H	-0.479721	3.808309	2.990994

Dimer 2

C	4.123283	0.293241	5.010422
C	2.827901	0.127800	5.386151
C	2.142978	1.377767	5.188737
C	3.088962	2.345400	4.651134
N	4.300912	1.593401	4.605615
C	1.847272	-0.882287	5.760737
N	0.639196	-0.126971	5.823237
C	0.822770	1.180325	5.445777
O	1.910356	-2.080292	5.941989
O	2.986525	3.490554	4.264376
O	-0.473051	0.032097	2.703108
C	0.695072	-0.136635	2.423058
C	1.737385	0.731615	1.893903
C	2.972968	-0.004994	1.857157
C	2.729874	-1.259029	2.320554
N	1.395627	-1.362828	2.628587
C	4.045636	0.909859	1.490337
N	3.343200	2.130797	1.267779
C	2.003848	2.019192	1.548784
O	5.251223	0.795414	1.415361
H	3.398571	-2.091041	2.475729
H	0.965120	-2.161644	3.065660
H	3.823533	2.997361	1.089831
H	1.355447	2.879536	1.503925
H	4.939630	-0.411084	4.979204
H	5.147077	1.961015	4.201407
H	-0.256032	-0.563903	5.967988
H	-0.013677	1.856105	5.366420

Dimer 3

N	-0.252009	-0.674145	-0.053183
C	-0.075515	-0.831515	1.303562
C	1.190597	-0.457646	1.620066
C	1.861418	-0.063461	0.409956
C	0.935956	-0.206323	-0.705757
C	2.113159	-0.296414	2.741406
N	3.287141	0.205044	2.092639
C	3.119788	0.334468	0.736920
O	2.025811	-0.504538	3.927089
O	1.016226	-0.008251	-1.896253
H	3.913525	0.707422	0.109140
H	4.110825	0.457689	2.612770
H	-1.069331	-0.946802	-0.573396
H	-0.873308	-1.194569	1.932907
N	0.420676	2.998843	-0.058699
C	0.590436	2.869354	1.296650
C	1.849217	3.267767	1.621473
C	2.517729	3.662456	0.410242
C	1.593223	3.501104	-0.709451
C	2.776648	3.410491	2.735489
N	3.963349	3.878574	2.080960
C	3.784410	4.036318	0.724520
O	2.698555	3.212166	3.926181
O	1.678202	3.709690	-1.895194
H	4.581110	4.399539	0.093843
H	4.781633	4.151172	2.599743
H	-0.404007	2.746560	-0.577345
H	-0.202017	2.496084	1.925817
